# Effect of Spatial Repellent Exposure on Dengue Vector Attraction to Oviposition Sites

**DOI:** 10.1371/journal.pntd.0004850

**Published:** 2016-07-18

**Authors:** Diane B. Choi, John P. Grieco, Charles S. Apperson, Coby Schal, Loganathan Ponnusamy, Dawn M. Wesson, Nicole L. Achee

**Affiliations:** 1 Department of Biological Sciences, University of Notre Dame, Notre Dame, Indiana, United States of America; 2 Department of Entomology, North Carolina State University, Raleigh, North Carolina, United States of America; 3 Department of Tropical Medicine, Tulane University, New Orleans, Louisiana, United States of America; Mahidol University, THAILAND

## Abstract

**Background:**

*Aedes aegypti* is a primary vector of dengue virus (DENV), the causative agent of dengue fever, an arthropod-borne disease of global importance. Although a vaccine has been recommended for prevention, current dengue prevention strategies rely on vector control. Recently, volatile pyrethroids—spatial repellents—have received interest as a novel delivery system for adult *Ae*. *aegypti* control. Understanding the full range of behavioral effects spatial repellents elicit in mosquito species will be critical to understanding the overall impact these products have on vector populations and will guide expectations of efficacy against DENV transmission.

**Methodology/Principal Findings:**

The current study quantified changes in attraction of gravid *Ae*. *aegypti* to experimental oviposition sites following exposure to the spatial repellent transfluthrin. Responses were measured with two-choice olfaction bioassays using ‘sticky-screens’ covering cups to prevent contact with the oviposition substrate. Two cups contained a bacterial attractant composed of four species of bacteria in calcium alginate beads in water and two cups contained only deionized water. Results from 40 replicates (*n* = 780 females total per treatment) indicated an estimated difference in attraction of 9.35% ± 0.18 (p ≤ 0.003), implying that the transfluthrin-exposed mosquitoes were more attracted to the experimental oviposition sites than the non-exposed mosquitoes.

**Conclusions/Significance:**

Findings from this study will further characterize the role of spatial repellents to modify *Ae*. *aegypti* behavior related to dengue prevention specifically, and encourage innovation in vector control product development more broadly.

## Author Summary

As the incidence of dengue continues to expand, there is need for improved and/or novel strategies for vector control. This includes the use of spatial repellent products that contain volatile chemicals at ambient temperatures. When these chemicals are applied at low concentrations, they can serve to ‘repel’ rather than ‘kill.’ This study aimed to understand the effects of a spatial repellent, transfluthrin, on *Aedes aegypti* attraction to experimental oviposition sites in an effort to further understand how spatial repellents may impact vector populations.

## Introduction

Dengue virus (DENV) is the causative agent of dengue, an arthropod-borne disease of global burden endemic in over 100 countries where an estimated 2.5 billion people live [1]. There are four DENV serotypes (DENV1 to 4). Each serotype causes a range of disease in humans, from asymptomatic to mild fever and the more severe, hemorrhagic and shock syndrome manifestations. Although the World Health Organization recently recommended the use of the CYD-TDV dengue vaccine [2, 3], currently there is no preventative therapy or curative treatment for dengue fever; mitigation of symptoms is supportive and prevention relies greatly on vector control, much like many other arthropod-borne diseases [1, 4]. There are multiple vector control strategies recommended by the World Health Organization for both immature and adult *Aedes aegypti* life stages [1]. These include biological agents and synthetic chemicals that are used to treat mosquito production sites as well as the interior and exterior areas of houses [1].

Given the lack of indication that new vector control chemicals will be available soon [4] and the dependency of a small range of synthetic chemicals in currently existing products, it has become increasingly important to understand the wider effects of these active ingredients on individual mosquito behavior, and populations as a whole, in relation to risk of pathogen transmission [4, 5]. Previous studies have demonstrated that chemicals currently recommended for vector control can elicit varied responses dependent on primary mode of action [6] as well as concentration [7]. For instance, some pyrethroids, such as deltamethrin are applied at predominately toxic levels to kill mosquitoes that land on treated surfaces with residual effects lasting months following a single application. Others, such as transfluthrin and metofluthrin, labeled as spatial repellents, are highly volatile at ambient temperature and serve to repel, as well as kill, mosquitoes due to a concentration gradient in the air space that flying insects encounter [8–10]. Today, all long-lasting insecticide-treated bed nets (LLINs), ultra-low volume (ULV) sprays, and more than 80% of indoor residual spraying (IRS) include pyrethroids [4]. It is clear the residual pyrethroids used in insecticide-treated bed nets (ITNs) has contributed to reducing deaths caused by malaria, another mosquito-borne disease [11]. However the efficacy of these chemicals is threatened by increasing levels of pyrethroid resistance found in several areas of the world such as West Africa where some mosquito populations express more than 1000-fold resistance, compromising the efficacy of IRS and LLINs [4]. In addition, although effective against malaria, ITNs are not as useful for dengue prevention as *Ae*. *aegypti* biting occurs during day-time periods when people are typically not sleeping and therefore not under nets [12]. It is due to this day-biting behavior, and potential for volatile pyrethroids to repel vectors from human contact, that spatial repellents have been gaining wider attention as a potential malaria [13–15] and DENV transmission breaking strategy [9].

Spatial repellent products are intended to work by releasing chemicals into the air to reduce mosquito entry into a treated space, and inhibit host-attraction and/or blood-feeding on humans [16]. Experimental hut studies have reported a wide range of anopheline mosquito behaviors affected by airborne pyrethroids such as blood feeding inhibition, deterrence, increased contact irritancy and excito-repellency as well as reduced fecundity [17–19]. It is evident that these volatile semiochemicals are able to modify epidemiologically relevant behaviors that have the potential to disrupt transmission dynamics. However, the full extent of mosquito behaviors elicited and/or modified by spatial repellents is still unclear. A better understanding of behavioral effects of spatial repellents on disease vectors is critical for defining expectations of protective efficacy against pathogen transmission post-exposure to the repellent [20–22]. With this in mind, the specific objective of the current study was to quantify effects of transfluthrin exposure of gravid *Ae*. *aegypti* on attraction to experimental oviposition sites under laboratory conditions.

## Materials and Methods

**Fig 1:**
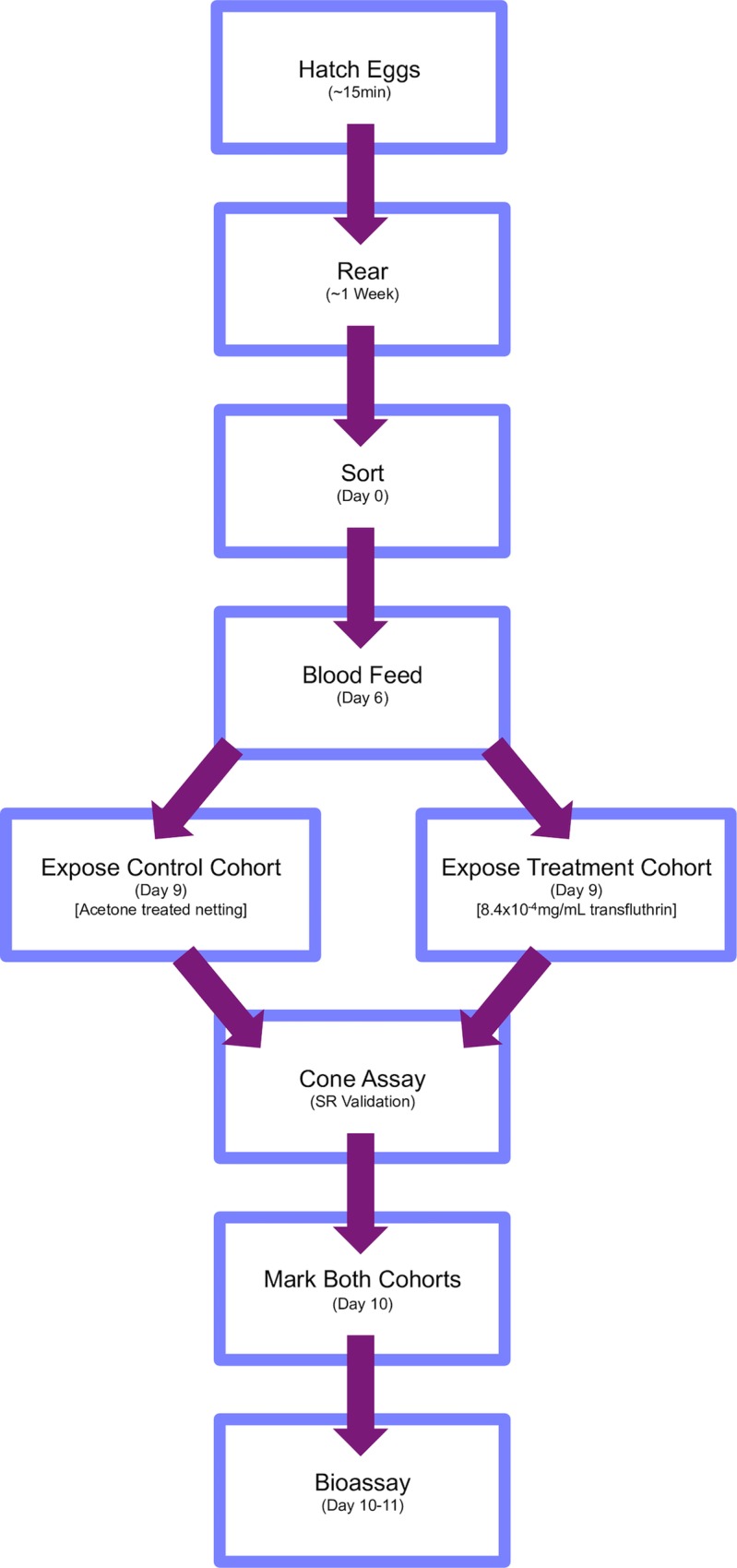
Overall study design of exposure experiments.

Fig 1 outlines the overall study design of exposure experiments.

### Mosquito cohorts

*Aedes aegypti* colonies were established from larvae collected in June 2015 in Orange Walk, Belize, Central America as part of routine Ministry of Health dengue vector surveillance activities. These populations have been characterized as pyrethroid susceptible [18]. Eggs representing generation F1 were provided to the University of Notre Dame laboratory for production of test cohorts. Larvae were reared to adults using previously described protocols [23]. Six days post-eclosion cohorts of 300 females were fed to repletion on human blood purchased through a blood bank (Interstate Blood Bank, Inc., Memphis, TN) with added ATP, a 5mM solution consisting of 0.025g per 10 mL blood (Sigma-Aldrich, St. Louis, MO) using a membrane feeding system (approx. 60min) and then maintained at 28°C and 80% RH prior to spatial repellent exposure.

### Transfluthrin exposure

**Fig 2:**
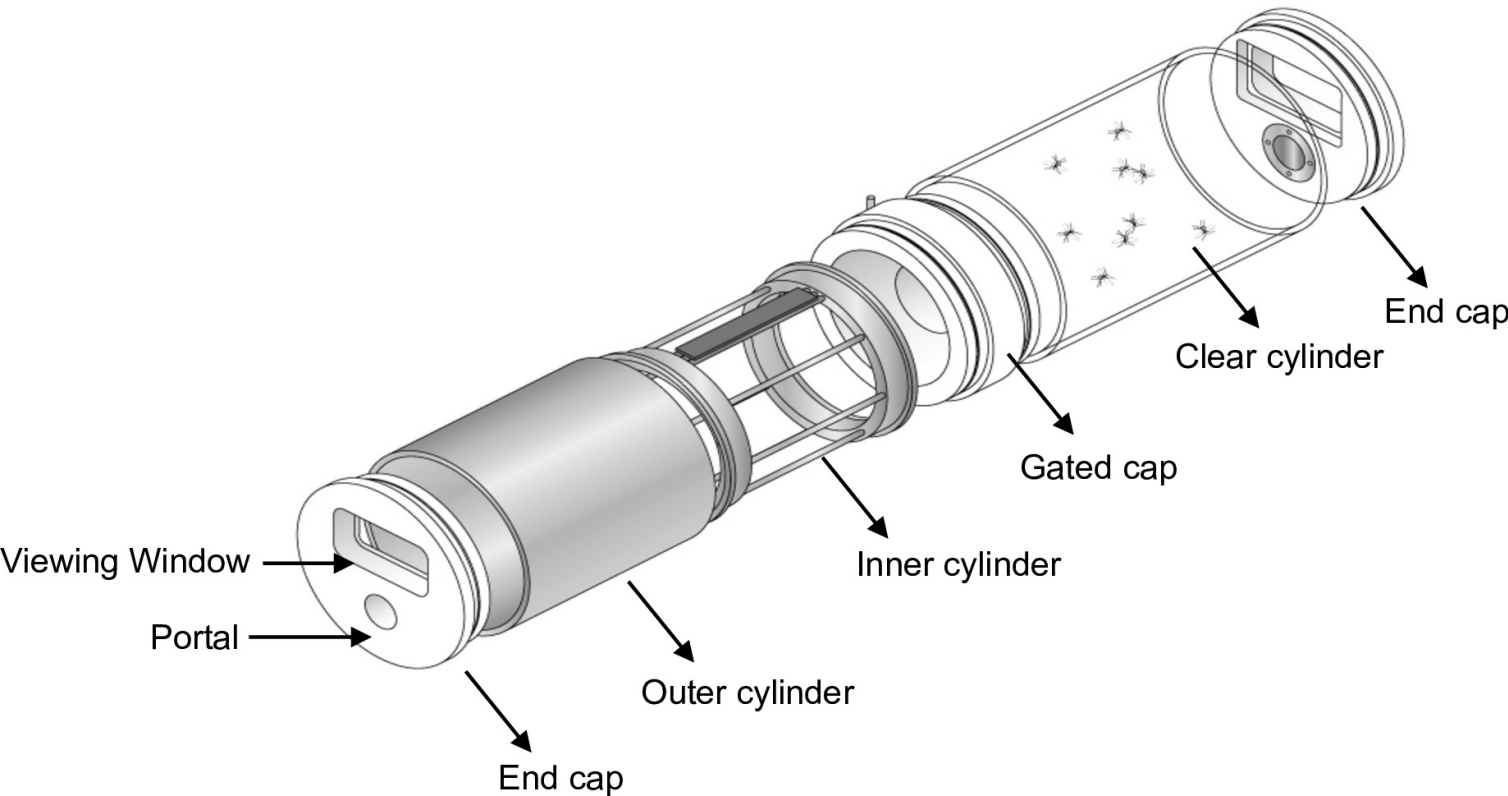
High-Throughput Screening System (HITSS). The HITSS chambers are modular, consisting of metal cylinders attached to clear cylinders. Treated netting is placed inside the metal cylinder and mosquitoes introduced into the clear chamber to prevent making direct contact with the chemical but allowing for chemical exposure (by permission of the American Mosquito Control Association) [25].

At 3 days post-blood feeding, groups of 10 *Ae*. *aegypti* females were exposed to either transfluthrin solution (in acetone) ata concentration of 8.4x10^-4^ mg/mL (treatment) or acetone only (solvent control) at a time using the High-Throughput Screening System (HITSS) (Fig 2) and previously described protocols [18, 24]. This transfluthrin concentration was based on a validated repellent response of *Ae*. *aegypti* (Belize strain) to transfluthrin in previous dose-response studies [18]. Specifically, 1.5mL of solution was applied to 11cm x 25cm nylon organdy netting strips (No. I10N, G-Street Fabrics, Bethesda, MD), corresponding to 4.6 ng/cm^2^, allowed to air dry for 15min then placed into individual metal chambers of the HITSS assay [25]. Mosquitoes were transferred into clear chambers attached to the metal compartments holding the netting strips. This configuration prevented mosquito tarsal contact with treated netting and therefore mimicked the exposure route expected under field conditions (i.e., chemical dispersal in air). Groups of 10 female mosquitoes from the same cohort were placed in the chamber for a 10 min exposure [24]. The 10 min exposure time was based on previous spatial repellent studies using the HITSS system [6, 18, 25]. After the 10 min exposure, cohorts were released into individual cages depending on treatment, collected and placed into holding containers then marked with fluorescent pigment powder (blue for transfluthrin exposed and white for control cohorts; DayGlo, Rancho Dominguez, CA). Together, the exposure and marking process took approximately 30 min per group of 10 mosquitoes. After marking, the cohorts were held for 24hr at 28°C and 80% RH to monitor mortality prior to conducting attractant bioassays. The total number of mosquitoes introduced into the bioassay test arenas varied by trial due to mosquito availability; however, each trial consistently included equal numbers of exposed and unexposed cohorts (Table 1).

**Table 1:** Sample size of *Aedes aegypti*^1^ (exposed and unexposed)^2^ observed during study.

Trial	No. Replicates (Arenas)	No. per Cohort/Arena	Total No. per Cohort/Trial	Total No. Observed/Trial
1	4	10	40	80
2	4	25	100	200
3–10	4	20	640	1280
**Grand Total**	40		780	1560

^1^Belize strain, F2, 10 days old, blood fed 6 days post-eclosion; exposed 24hr prior to choice bioassay for 10 min, 24hr pre-test

^2^exposed to either 4.6ng/cm^2^ transfluthrin or solvent only for 10min in the HITSS system

### Bioassay test arena

**Fig 3:**
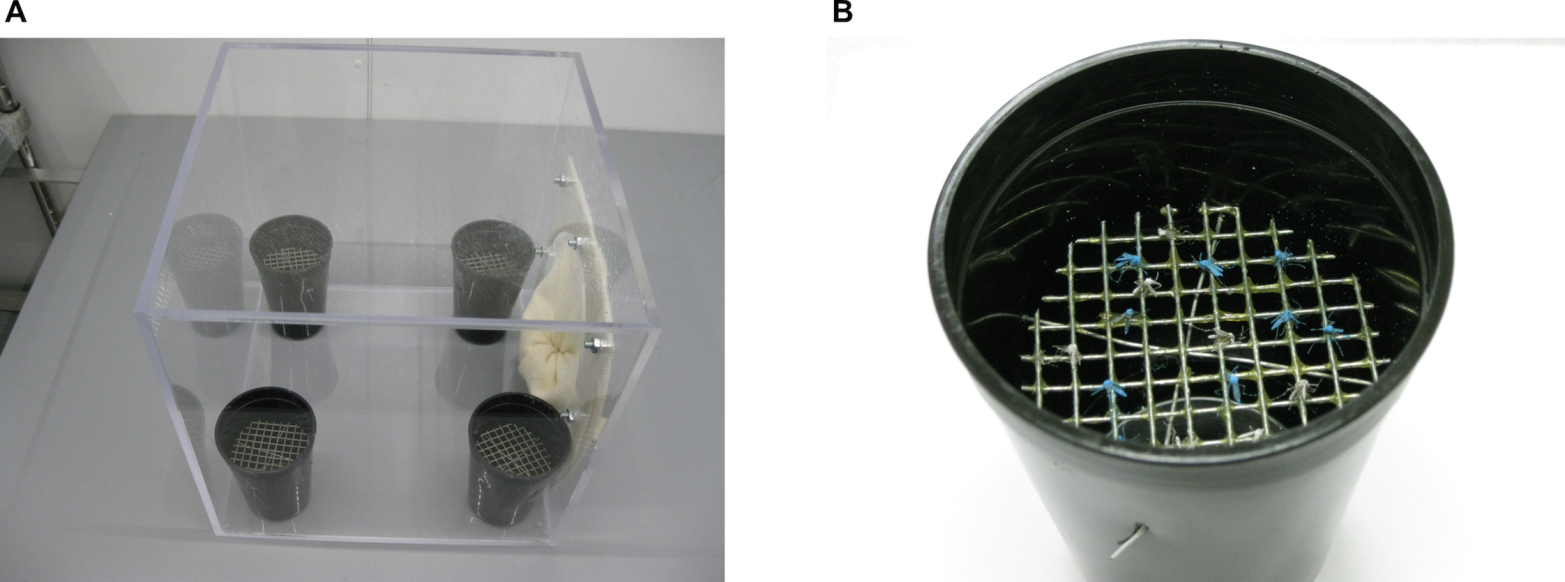
**A. Bioassay test arena.** Each bioassay test arena is composed of 30x30x30cm Plexiglas containing four experimental oviposition sites. **B. Experimental oviposition site.** Black plastic cup containing either attractant bacterial beads or water only (matched control) covered by a wire mesh ‘sticky-screen’ that captured pigment powder marked gravid *Ae*. *aegypti*.

Each of four 30x30x30cm Plexiglas bioassay test arenas (Fig 3A) had a total of four 11x8cm black plastic cups (WNA, Houston, TX), representing experimental oviposition sites. Two cups were filled with 30mL deionized water (control) and the other two cups were filled with 30 mL of water containing calcium alginate-formulated beads that include a mix of 4 bacterial isolates previously shown to attract gravid *Aedes* [26, 27]. The attractant formulation was prepared by placing 0.1g of the bacterial beads in 500mL deionized water and this suspension was stored at 4°C until use 14–35 days later to ensure bacterial dispersion in the suspension. Just before use, the bacterial suspension was diluted 1:1 with water. Each cup had a ‘sticky-screen’ covering, consisting of wire mesh (0.25x0.25cm) dipped in insect glue (Tanglefoot, Tangle Foot Co., Grand Rapids, MI) prepared as previously described [26, 28] (Fig 3B). Wire mesh pieces were placed 4.2cm below the cup rims so that the position was a standard height above the surface of the water. At 2PM on each day of testing, cups were prepared and positioned in the bioassay test arenas and marked females from each transfluthrin-exposed and unexposed cohorts were introduced for a 24hr period.

After the 24hr testing period, a microscope was used to count the number of mosquitoes captured on each sticky-screen by observing the pigment in order to identify the treatment group. Data on the sticky-screen captured mosquitoes was recorded for each cup position in all of the bioassay test arenas. Those knocked-down on the arena floor were counted using the same identification methods. Mosquitoes that were flying at the completion of the bioassay were counted, collected and held for an additional 24hr to record ‘48hr post-exposure mortality.’ Four replicates (bioassay test arenas) were conducted simultaneously on a single day of testing, together comprising one trial. Ten trials were conducted throughout the entire study, resulting in a total of 40 replicates with a total *n* = 780 females for each of the cohorts, unexposed and transfluthrin-exposed (Table 1). All bioassays were conducted in a testing room maintained at 25°C and 80% RH.

### Chemical validation

An additional bioassay was performed to confirm transfluthrin presence on HITSS netting. Standard WHO cone bioassays were conducted per protocol [16] using netting strips (both treatment and control) from HITSS exposure procedures immediately after removing mosquitoes from the system. Two plastic cones were pinned over a 3x3cm area of each of the netting strips positioned on a metal test board set at a 45° angle. Five gravid *Ae*. *aeygpti* females that were not used in the HITSS, but were maintained and blood-fed under matched conditions of test cohorts, were introduced into each cone and held for 3 minutes. After the holding period, knock-down was immediately recorded, then mosquitoes were transferred to individual cartons and maintained at 28°C and 80% RH for an additional 24hr to record 48hr post-exposure mortality. A total of 20 cone bioassays were performed per netting strip.

### Statistical analysis

Attraction of gravid female mosquitoes to experimental oviposition sites was quantified using the Oviposition Activity Index (OAI), which is equal to [Nt-Nc]/[Nt+Nc] where *Nt* is the number of gravid mosquitoes trapped on the sticky-screen of the treatment cups and *Nc* is the number of gravid mosquitoes trapped on the sticky-screen of the control cups [29]. The OAI ranges from -1 to +1 with 0 indicating no difference in attraction between treatments. A value closer to +1 indicates attraction to the bacterial beads, while a value closer to -1 indicates greater attraction to the water control. OAI values were calculated to confirm the presence of the attractant in the treatment cups. Attraction differences between transfluthrin exposed and control cohorts were calculated by tabulating the number of individuals landing in cups containing the attractant formulation versus cups containing water only for both the exposed and non-exposed cohorts released in each bioassay test arena. Each count was then divided by the total number of mosquitoes released into each bioassay test arena per cohort. The difference between the exposed and non-exposed was taken to obtain one value per replicate conducted (*n* = 40). A two-tailed t-test was conducted using the mean of the attraction differences between cohorts to test for the significance using SPSS 23 software. Additionally, the Shapiro-Wilk test for normality was used to confirm that the data were normally distributed (p>0.05) prior to applying t-test statistics.

## Results

Results from WHO cone bioassays indicated a knock-down rate of 39.8% (*n* = 159/400) and 15.3% 24hr mortality (*n* = 61/400) in mosquito populations evaluated against the transfluthrin treated netting. By contrast, no knock-down (KD) or mortality was observed in cohorts placed in the HITSS assay containing the same transfluthrin nettings. This confirmed that mosquitoes were exposed to a non-toxic concentration of transfluthrin in the HITSS air space. There was 0% (*n* = 0/400) knock-down and 0% (*n* = 0/400) mortality exhibited in test cohorts from cone bioassays using HITSS netting strips treated with acetone solvent only and no knock-down or mortality was observed during the HITSS exposure to control netting.

Mosquitoes exposed to transfluthrin were more likely to land in containers within a given bioassay arena (8.18 ± 0.52 mosquitoes) than non-exposed cohorts (5.35 ± 0.43 mosquitoes). Additionally, calculated OAI values from choice bioassay data indicated transfluthrin-exposed gravid *Ae*. *aegypti* mosquitoes were more attracted to sites containing the bacterial beads than to cups containing water only (OAI: 0.363). Conversely, while the OAI value of non-exposed females also indicated attraction to the bacterial beads, the response was weaker (OAI: 0.118). Specifically, unexposed gravid *Ae*. *aegypti* were 22% more likely to be attracted to containers with the bacterial attractant beads compared to water, whereas mosquitoes that were pre-exposed to transfluthrin exhibited greater attraction (30.0%; *n* = 234) to the bacteria compared to unexposed females in the same bioassay test arenas (14.5%, *n* = 113; Table 2). The mean difference in attraction was significant (9.35% ± 0.1855; p ≤ 0.003). There was no observed knock-down after HITSS exposure in either test population and no difference in mortality prior to bioassays between exposed and non-exposed cohorts (Table 2).

**Table 2:** Effects of transfluthrin (n = 780) or solvent (n = 780) exposure on gravid *Ae. aegypti*^1^ attraction to experimental oviposition sites.

	Transfluthrin Exposed		Solvent Exposed	
Observed Behavior	Percent (%)	n/total	Percent (%)	n/total
Attraction to bacterial beads^2^	30.0	234/780	14.5	113/780
Attraction to control	15.0	117/780	11.9	93/780
Flying/Resting^3^	50.0	390/780	70.1	547/780
Knock-down^4^	5.00	39/780	3.46	27/780
48hr mortality^5^	5.89	23/390*	4.20	23/547*

^1^Belize strain, F2, 10 days old, blood fed 6 days post-eclosion; exposed for 10 min, 24hr pre-test

^2^30mL of bacterial bead (0.1g per 500mL,14–35) diluted at 50% in water [26]

^3^Flying/resting counted after 24hr bioassay

^4^ Immediately after completion of bioassay; defined as moribund or dead [16]

^5^ Mortality in flying/resting populations after 24hr maintenance post-bioassay, 48hr post-exposure

*Mortality percent is calculated where (n/(flying or resting))

## Discussion

The objective of the current study was to quantify the effect of a volatile spatial repellent, transfluthrin, on *Ae*. *aegypti* attraction to experimental oviposition sites under laboratory conditions. Although the World Health Organization just recently recommended the CYD-TDV vaccine for dengue prevention [3], vector control will continue to be a vital component of disease prevention in an integrated system [1, 15]. Exploiting *Aedes aegypti* preference for, and interruption of attraction to, an oviposition site can have a direct impact on the likelihood of survival for subsequent DENV transmission [30, 31].

Overall, findings reported here demonstrate a significant increase in attraction response of gravid *Ae*. *aegypti* females to experimental oviposition sites following exposure to transfluthrin in the volatile phase as compared to non-exposed cohorts. The mechanism of action responsible for this increased attraction is unclear. This behavioral change may be due to a hyperactive olfactory response of gravid females elicited by the spatial repellent, a hypothesis that needs further exploring using electrophysiological methodologies [32]. Indeed, the data support a heightened sensory mechanism of action as there was an increase in the number of transfluthrin-exposed mosquitoes landing in oviposition containers overall and fewer mosquitoes flying as compared to unexposed *Ae*. *aegypti*. Heightened olfactory acuity can result from extensive grooming, especially of the antennae [33]. It is possible that the transfluthrin-exposed mosquitoes had increased levels of antennal grooming, which may have caused insects to have more acute olfaction; additional studies should include video observations to explore grooming behavior after exposure to spatial repellents. The sublethal concentration of transfluthrin may have also induced a hormetic effect on test mosquitoes. Hormesis is a phenomenon where a positive effect occurs in response to exposure to low doses of a chemical that normally causes negative effects at higher doses [34]. A hormetic effect has previously been demonstrated in pest insects exposed to deltamethrin in relation to increased breeding propensity [35, 36]. In the context of *Aedes aegypti* and transfluthrin, a similar hormetic effect may explain why there was a greater attraction to the experimental oviposition sites. Further studies are needed to confirm this hypothesis.

Despite currently unknown mechanism of action, the demonstration of enhanced attraction response of transfluthrin-exposed mosquitoes may offer insights into a complementary, or perhaps synergistic, role for spatial repellents in combination with lethal attractant gravid traps (a push-pull strategy) in dengue endemic settings. The use of spatial repellents in combination with adult mosquito traps has been previously proposed and explored [9, 18, 37] but the public health value is still unknown until epidemiological evidence is generated. Likewise, transfluthrin exposure to *Ae*. *aegypti* may alter the natural ‘skip-oviposition’ phenomenon observed by this species [38], thereby enhancing the use of ovitraps as a single intervention [30]. These and other operational research studies are warranted. Previous studies evaluating transfluthrin coil exposure of *Anopheles gambiae sensu lato* reduced human vector contact through deterrence by 38% and induced approximately 56% of the mosquitoes to leave the hut before feeding [17]. Although it is challenging to know what a 9.35% difference in attraction to oviposition sites translates to in population dynamics, the use of transfluthrin is likely to impact various mosquito behaviors (oviposition attraction, deterrence, feeding), which can reduce the probability of human-vector contact.

The authors recognize limitations in the data generated that may challenge translation of findings to field conditions. First, transfluthrin is one of six currently registered spatial repellents [10, 39]. Although these are all pyrethroids each may elicit potentially different behavioral effects. Likewise, it is unknown if similar behavior responses would be seen using different chemical classes. Additional experiments should include *Ae*. *aegypti* populations with various insecticide resistance profiles and both pyrethroid resistant and tolerant vector populations, especially in light of the global burden of reported *Ae*. *aegypti* insecticide resistance that challenges the use of pyrethroid chemicals for dengue vector control [40]. Indeed, recent studies exploring the hereditability of spatial repellent behavior have shown an attenuated repellent response linked to target site resistance mechanisms [13]; although the attenuation could be reversed in a single generation. Similar evaluations used in the current study should also be applied against *Ae*. *albopictus* to better understand spatial repellent secondary effects in dengue endemic settings where this vector is responsible for transmission [41]. Perhaps most challenging is translating a 9.35% increase in attraction of transfluthrin-exposed *Ae*. *aegypti* observed under laboratory conditions on population dynamics within natural environments. This is unclear but is the authors’ intent that data from this and ongoing studies exploring vector behaviors that may be influenced by spatial repellent exposure be applied in models related to egg-laying and/or oviposition-site choice to help understand and predict population-level changes in field settings [31, 42–44].

More important to the objective of this study, findings have advanced our understanding of the range of effects spatial repellent chemicals have on mosquito behavior, thereby providing valuable information to consider in the development of vector control chemicals. Specifically, results support a broader functionality of volatile repellents beyond their current application (to prevent human biting) that could facilitate expanding label uses of available products. It is hoped this may further incentivize discovery, development and evaluation of new spatial repellent strategies for dengue and other arthropod-borne disease prevention.
